# Inhibitory Effect of *Berberis vulgaris* Aqueous Extract on Acquisition and Reinstatement Effects of Morphine in Conditioned Place Preferences (CPP) in Mice

**DOI:** 10.17795/jjnpp-16145

**Published:** 2014-06-16

**Authors:** Mohsen Imenshahidi, Reza Qaredashi, Mahmoud Hashemzaei, Hossein Hosseinzadeh

**Affiliations:** 1 Department of Pharmacodynamy and Toxicology, Pharmaceutical Research Center, School of Pharmacy, Mashhad University of Medical Sciences, Mashhad, IR Iran; 2 Department of Pharmacodynamy and Toxicology, School of Pharmacy, Mashhad University of Medical Sciences, Mashhad, IR Iran; 3 Department of Pharmacology and Toxicology, School of Pharmacy, Zabol University of Medical Sciences, Zabol, IR Iran; 4 Pharmaceutical Research Center, School of Pharmacy, Mashhad University of Medical Sciences, Mashhad, IR Iran

**Keywords:** *Berberis vulgaris*, Withdrawal Syndrome, Morphine, Methods

## Abstract

**Background:**

It has been elucidated that *Berberis vulgaris* (barberry) can alleviate morphine withdrawal syndrome. Also it has been reported that aqueous extract of barberry possibly have inhibitory effect on NMDA receptors.

**Objectives:**

In this study, we decided to evaluate the effects of aqueous extract of *B. vulgaris* fruit on morphine tendency in mice using conditioned place preference (CPP) method.

**Materials and Methods:**

In experiment 1 (acquisition phase), mice underwent morphine-induced conditioned place preference (CPP) training with injections of morphine (40 mg/kg). In experiment 2 (extinction and reinstatement phases), mice underwent the same CPP training as in experiment 1 and subsequent extinction training on day 16th a reinstatement by CPP was done by injection of reminding 10 mg/kg morphine.

**Results:**

The administration of morphine (40 mg/kg for four days) produced place preference. In the first method, the aqueous extract of barberry (200 mg/kg) prevented morphine tendency to white cell in CPP method. In the second method, after inter-peritoneal injection of aqueous extracts of barberry at 100 and 200 mg/kg, the animals tendency toward the white cells of CPP chamber on the sixteenth day (after a reminder injection of morphine 10 mg/kg) was significantly reduced.

**Conclusions:**

These results show that aqueous extract of barberry can reduce the acquisition and reinstatement of morphine-induced conditioned place preference.

## 1. Background

Relapsing to opioid after abstinence therapy is a major clinical problem in addicted people who were undergoing detoxification (1). Drug desiring is a subjective feeling which motivates to drug seeking and can produce relapse to drug abuse even long-time after withdrawal. It has clarified that the dopamine mesolimbic (DA) system is the principal pathway that can cause opioids’ psychological dependency (2). Increasing the activity of this system is accompanied by euphoria feeling, which leads to continuing drug abuse (3). Many studies have revealed the mesocorticolimbic DA system contribution to the acute reinforcing effects of opioids (4-6). DA neurons in the ventral tegmental area (VTA) activated by opiates, inhibit GABAnergic inhibitory interneurons, which increase the DA transmission to the nucleus accumbens (NAcc) (7). Some dopamine antagonists like haloperidol, clozapine, risperidone and SCH 23390 have opposed morphine-induced conditioned place preference (CPP) in mice (4). It is supposed that it may not be the unique way of opioids rewarding system. The VTA and the NAcc received glutamatergic projections from the prefrontal cortex (PFC) and limbic areas. Biochemical studies have demonstrated the regulation of DA release by glutamate and N-methyl-D-aspartate (NMDA) receptors (7). NMDA receptor antagonist, i.e. memantine, can inhibit acquisition of morphine induced CPP (8). Barberry (*Berberis vulgaris* L. from the family of Berberidaceae) grows in Asia and Europe; the plant is well known in Iran and has been used traditionally as a medicinal plant. The fruits of the plant have been also used as a food additive. *B. vulgaris* L. is a spiny, yellow wood and obovate leaves, plant, up to 3 meter tall with bearing pendulous yellow flowers succeeded by oblong red fruits (barberry) (9, 10). Its fruits have many indications such as treatment of ailments and discomforts of kidneys, urinary, gastrointestinal tract, liver diseases, bronchial discomforts, and as a stimulant of the circulatory system (11).

Some plants and their constituents such as *Salvia leriifolia* (12), *Rosmarinus officinalis* (13), *Crocus sativus* (14), *Berberis vulgaris*, (15) linalool (16), *Trachyspermum copticum* (17), *Zhumeria majdae* (18), *Stachys byzantine *(19), *Ferula gummosa* (20), *Valeriana officinalis* (21) and *Mentha longifolia* (22) reduced signs of withdrawal syndrome in animals studies. Many studies on reinforcing effects with high dependence likelihood were widely evaluated by the conditioned place preference (CPP) method (23). We showed that saffron and its constituent, crocin, can reduce the acquisition and reinstatement of morphine-induced conditioned place preference (24, 25).

## 2. Objectives

*B. vulgaris* fruit extract reduced morphine dependence (15) and showed inhibitory effect on NMDA receptors (26). Based on these evidences, we want to clarify whether *B. vulgaris* fruit extract (BVFE) could reduce morphine tendency in mice using CPP method. In this study, the effect of aqueous extract of barberry on acquisition and reinstatement of morphine-induced CPP was evaluated in mice.

## 3. Materials and Methods

### 3.1. Animals

Male NMRI mice (25-30 g) were kept under standard conditions (free access to food and water), at 25˚C, 12 hours light/dark cycle. All animals were treated in accordance with the guidelines for the care and use of laboratory animals prepared by the Animal Care Committee of Mashhad University of Medical Sciences.

### 3.2. Preparation of Extract

*Berberis vulgaris* was collected from Gonabad (a town in southern Razavi Khorasan province) and authenticated by the Herbarium of Pharmacy School of Mashhad University of Medical Sciences, IR Iran (Voucher No: 1542). The fruits were powdered by grinder mixer after drying under shade. Then, the powder was extracted using aqueous decoction method. In this method, 100 g of the powdered fruits was added to 1 liter of boiling water for 15 minutes and then filtered through a cloth. The extract was then concentrated under reduced pressure to the desired volume (15). The yield of the extract was 10% (w/w).

### 3.3. Chemicals

The following reagent was used: morphine sulphate (Darou Pakhsh, IR Iran).

### 3.4. Apparatus

The apparatus was composed of three different compartments, made of Plexiglas. Two chambers of the same size (30 length × 30 width × 35 cm height) were connected by the third chamber (15 length × 30 width × 35 cm height). Chambers walls had different colors (black vs. white) with distinct floor textures (fine and wide grid in the black and white compartment, respectively).The olfactory cues are banana extract from which a drop was placed at the corner of the black compartment floor and a drop of acetic acid was placed at the corner of the white compartment floor. In order to prevent the interference of odor produced by feces and/or urine, whole box were cleaned for each test.

### 3.5. Experimental Procedure

#### 3.5.1. Acquisition of Place Preference

##### 3.5.1.1. Pre-Conditioning Phase

The study had three phases. The first phase lasted for 3 days. In the first two days, animals were allowed to move freely in each chamber for 15 minutes. On the third day, the times that they spend in each black or white chamber were recorded. The mouse which spent more than 500 sec in the white chambers or more than 400 sec in the central grey compartment was excluded from the test (24, 27)

##### 3.5.1.2. Conditioning Phase

This phase lasted for 4 days. Animals received a single intra-peritoneal normal saline and were placed for an hour in the black chamber of CPP. After 4 hours, they received either morphine or BVF, intra-peritoneally. After treatment (morphine or BVFE), they were placed for one hour inside the white chamber of PCC. Animals were divided into 8 groups (n = 7): saline + saline (SAL); saline + morphine 40 mg/kg (MOR); morphine 40 mg/kg + BVFE extract 50, 100, 200 mg/kg, (MOR + EXT 50, 100 and 200); saline + BVFE extract 50, 100, 200 mg/kg (EXT 50, 100 and 200). Immediately following drug administration, animal were placed in the white compartment for one hour.

##### 3.5.1.3. Post-Conditioning Phase

During the third phase (post-conditioning), on the eighth day of study, mice were placed into the apparatus without any barrier between chambers, where they were able to move freely between the chambers (guillotine doors were removed). The total time that every mouse spent on any chambers were calculated for 15 minutes. The time spent in the central area was proportionally divided between both conditioning compartments. The time each mouse spends in the middle chambers is divided between two chambers equally. The time each mouse spent in the white and black chambers is calculated as follows: time spends during pre-conditioning phase-time spent during post-conditioning phase. If the result is a positive number, it means that the drug has induced a preference and vice versa (24, 27).

#### 3.5.2. Extinction of Place Preference

After the first step of this study, the second step, the effects of BVFE on the morphine-induced CPP reinforcement were evaluated. Three phases of pre-conditioning, conditioning and post-conditioning were done as described above. In order to eliminate morphine tendency, after these three phases, animals were placed into the CPP chamber 60 minutes daily for 7 days. In order to induce morphine reinforcement, animals were placed inside the chamber for 900 sec on the 16th day, and the total time spent in the white and black chambers was calculated. Thirty minutes after drugs administration in each group, morphine 10 mg/kg plus normal saline (morphine group), morphine 10 mg/kg + BVFE 50, 100 and 200 mg/kg (EXT 50, 100 and 200 groups) were injected. For this purpose, animals were experienced in a 15 minutes daily extinction session schedule, which was consisted of placement of animals in the apparatus (without guillotine doors separating the compartments) at 8th day so that the time spent in the white compartment for each group of animals became similar to those of pre-conditioning sessions (24, 27).

#### 3.5.3. Reinstatement of Place Preference

Inducing the reinstatement by CPP was done by injection of reminding 10 mg/kg morphine, after the last day of extinction period (16th day). Thirty minutes before the first morphine injection, animals were injected morphine and BVFE (50, 100 and 100 mg/kg). After 30 minutes, each mouse was placed into the CPP chambers for 15 minutes and calculated the time spent in black and white chambers, was calculated (24, 27).

### 3.6. Statistical Analysis

Data are expressed as mean ± SEM. Statistical analysis was performed using one-way ANOVA followed by the Tukey-Kramer post-hoc test for multiple comparisons. P values less than 0.05 were considered statistically significant.

## 4. Results

### 4.1. Effects of B. vulgaris Fruit Extract on the Morphine Acquisition of Place Preference Test

**Figure 1.**
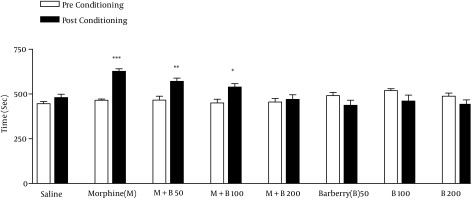
Effects of *Berberis vulgaris* Fruit Extracts on Mzorphine-Induced Conditioned Place Preferences

After repeated treatment with morphine, mice acquired CPP on day 8 (Figure 1). The administration of BVFEs (50, 100 and 200 mg/kg) were not induced place preference or aversion by itself. BVFE at dose of 200 mg/kg inhibited CPP (Figure 1).

During the phase of conditioning, animals received the following treatments in the drug-paired compartment: SAL, saline plus saline; MOR, saline plus 40 mg/kg of morphine; MOR + B50, 50 mg/kg of barberry plus 40 mg/kg of morphine; MOR + B100, 100 mg/kg of barberry plus 40 mg/kg of morphine; MOR + B200, 200 mg/kg of barberry plus 40 mg/kg of morphine; B50, 50 mg/kg of barberry plus saline; B100, 100 mg/kg of barberry plus saline; B200, 200 mg/kg of barberry plus saline. The bars represent the time spent in the drug-paired compartment before conditioning sessions in pre-conditioning test (white bars) and after conditioning sessions in post-conditioning test (black bars). *** P < 0.001, ** P < 0.01, * P < 0.05 significant difference in the time spent in the drug-paired compartment in pre-conditioning vs. post-conditioning sessions tests.

After acquisition and extinction of morphine-induced CPP, during reinstatement phase, animals received priming dose of morphine as following treatments: MOR + SAL, morphine (10 mg/kg) plus saline; MOR + B50, morphine (10 mg/kg) plus barberry (50 mg/kg); MOR + B100, morphine (10 mg/kg) plus barberry (100 mg/kg); MOR + B200, morphine (10 mg/kg) plus barberry (200 mg/kg). The bars represent the mean (± SEM) time spent in the drug-paired (white) compartment before conditioning sessions (white bars), after conditioning sessions (black bars), in the last extinction session (dark grey bars) and in the reinstatement test (light grey bars). *** P < 0.001, ** P < 0.01, *P < 0.05 significant difference in the time spent in pre-conditioning vs. post-conditioning sessions or reinstatement tests.

### 4.2. Effects of B. vulgaris Fruit Extract on Reinstatement of Place Preference

**Figure 2.**
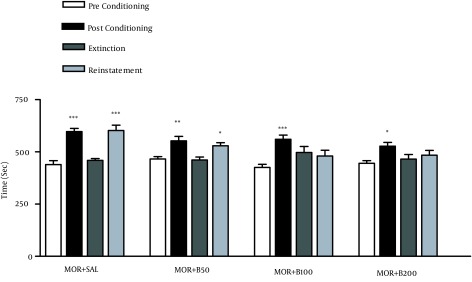
Effects of *Berberis vulgaris* Fruit Extracts on the Reinstatement of Morphine Induced Conditioned Place Preferences

No significant difference was found between the last extinction sessions (extinction) and pre-conditioning phases after daily extinction sessions; therefore, the conditioning has been disappeared. The administration of the priming dose of morphine (10 mg/kg) reinstated CPP. BVFE in doses 100 and 200 mg/kg inhibited the reinstatement of place preference due to priming dose of morphine (Figure 2).

## 5. Discussion

Our results showed that the aqueous extract of barberry could decrease morphine conditional preference (CPP). Meanwhile, it was observed that aqueous extract of barberry does not create a mental tendency or repulsion by itself. In the second method, namely morphine reinforcement test, the aqueous extract of barberry has prevented morphine reinforcement that was induced by injection of reminding morphine single dose on the 16th day. Morphine CPP is one of the very complicated phenomenon, in which many mechanisms such as dopaminergic, gabanergic and opioid systems and many brain nucleolus such as ventral tegmental, accumbens nucleus and hippocampus are involved (28). It is likely that one of the mechanisms that BVFE exerted is the antagonistic effect on NMDA receptors (9). *B. vulgaris* fruit extract showed inhibitory effect on NMDA receptors (9) and the ethanol extract of *B. koreana* reduced the expression level of NMDA receptors (26), therefore, one of the mechanisms that may play a role in the effect of aqueous extract of barberry is the antagonistic effect on NMDA receptor. Berberine has antagonistic effect on the NMDA receptors, even though, the amount of barberine in the *B. vulagaris* is low (9). Previous studies showed that NMDA antagonists have prevented morphine tendency (7, 8). Memantine as NMDA antagonist can ameliorate morphine tendency in CPP method (8). Memantine in self-administration could attenuate morphine rewarding potential in mice (29). Popic et al. elucidated that systemic administration of a variety of NMDA receptor antagonists inhibits morphine's rewarding properties in the conditioned place preference test (3). They found that activation of NMDA receptors in the nucleus accumbens and ventral tegmental is necessary for elicitation of approach by environments, previously associated with morphine's rewarding action (3). Addictive effects of opioids can be imposed by their dopaminergic mesolimbic rewarding neurons effects that caused by dopamine releasing in the nucleus accumbens (30). It seems that euphoria was produced by dopamine releasing and leads to morphine reinforcement. Dopamine has an important role in the rewarding pathway and glutaminergic releasing neuron are present in nucleus accumbens and it was clarified that glutamate is a regulator of dopamine release (31). For instance, it has been reported that NMDA infusion into the nucleus accumbens leads to increased dopamine at that nucleus accumbens (32). It seems that glutaminergic pathways of nucleus accumbens has rules on the regulation of mesolimbic dopaminergic system and as well as abusing drugs such as morphine, which can be another mechanism of action that can be involved is dopaminergic antagonistic effect. Studies have revealed that dopamine antagonists can decrease morphine tendency at the CPP model (4). However, there is no study for evaluation of BVAE on dopaminergic receptors, but it seemed that anti-dopaminergic effect did not play any role in decreasing morphine tendency, because many studies have elucidated that dopamine antagonists do not have any effect on the CPP method (7). It means that they cannot reduce morphine reinforcement; whereas, in this study, BVAE was effective on both methods.

In conclusion, *B. vulgaris* aqueous extract could significantly reduce the acquisition and reinstatement of morphine-induced conditioned place preference. According to previous studies concerning interaction of the aqueous extract of *B. vulgaris* fruits with NMDA receptors, this effect of barberry on CPP may be due to inhibitory effets on NMDA receptors. The *B. vulgaris* fruits extract may have the potential to be effective in preventing relapse in morphine addicted individuals.
